# Water-Splitting Electrocatalysis in Acid Conditions Using Ruthenate-Iridate Pyrochlores**

**DOI:** 10.1002/anie.201406668

**Published:** 2014-09-04

**Authors:** Kripasindhu Sardar, Enrico Petrucco, Craig I Hiley, Jonathan D B Sharman, Peter P Wells, Andrea E Russell, Reza J Kashtiban, Jeremy Sloan, Richard I Walton

**Affiliations:** Department of Chemistry, University of WarwickCoventry, CV4 7AL (UK); Johnson Matthey Technology Centre, Sonning CommonReading RG4 9NH (UK); Research Complex at Harwell, Rutherford Appleton Laboratory, Harwell OxfordDidcot, Oxon, OX11 0FA (UK); Chemistry, University of SouthamptonSouthampton SO17 1BJ (UK); Department of Physics, University of WarwickCoventry, CV4 7AL (UK)

**Keywords:** electrochemistry, hydrothermal synthesis, iridium, ruthenium, X-ray absorption spectroscopy

## Abstract

The pyrochlore solid solution (Na_0.33_Ce_0.67_)_2_(Ir_1−*x*_Ru_*x*_)_2_O_7_ (0≤*x*≤1), containing B-site Ru^IV^ and Ir^IV^ is prepared by hydrothermal synthesis and used as a catalyst layer for electrochemical oxygen evolution from water at pH<7. The materials have atomically mixed Ru and Ir and their nanocrystalline form allows effective fabrication of electrode coatings with improved charge densities over a typical (Ru,Ir)O_2_ catalyst. An in situ study of the catalyst layers using XANES spectroscopy at the Ir L_III_ and Ru K edges shows that both Ru and Ir participate in redox chemistry at oxygen evolution conditions and that Ru is more active than Ir, being oxidized by almost one oxidation state at maximum applied potential, with no evidence for ruthenate or iridate in +6 or higher oxidation states.

The electrochemical splitting of water is of significant relevance for contemporary energy applications, playing a key role in applications such as water-splitting electrolyzers for hydrogen production, and in reversible fuel cells for clean electricity production.[1] Although a number of increasingly complex oxide phases have been synthesized for electrocatalysis at pH>7 that combine Earth-abundant elements,[2] catalysis under acidic conditions is desirable for various reasons; particularly due to the fact an acidic electrolyte has higher ionic conductivity and so offers high current densities, and also avoids the unfavorable formation of carbonates as contaminants, which readily occurs under alkaline conditions, reducing the lifetime of any device. Electrocatalysts that are able to withstand operating conditions at low pH and above ambient temperature are thus required and metallic oxides of ruthenium and iridium are established to be the most suitable for these stringent requirements.[1], [3] In acidic environments the anodic oxygen evolution reaction (OER) presents challenges in resource efficiency, in particular because high loadings of precious metal are typically used to provide sufficient durability and power economy. Rutile-structured RuO_2_ is presently the most active oxide catalyst for OER in acidic aqueous media; however, it is unstable at operational electrolysis potentials as it is believed to be oxidized to soluble RuO_4_.[4] IrO_2_ is more stable than RuO_2_, but is less active. To find catalysts with optimum activity and stability the use of mixed phases of IrO_2_ and RuO_2_, along with other rutile oxides, such as TiO_2_ or SnO_2_, or inert oxides, such as Ta_2_O_5_, have been investigated, both as polycrystalline powders and as films.[5]

The mixed-metal oxide materials so far used in acid OER electrocatalysis are not always atomically homogeneous solid solutions.[5] To investigate the scope for improved, high activity oxide materials for OER under acid conditions, we have used hydrothermal synthesis, primarily because this route permits the direct crystallization from solution of multielement oxide materials, without the need for annealing at elevated temperature to induce crystallization.[6] By this means we have now been able to prepare a series of conducting mixed ruthenium–iridium A_2_B_2_O_7_ pyrochlore materials with A=Na,Ce^IV^ and B=Ru^IV^,Ir^IV^ as nanocrystalline powders, based on the parent B=Ru^IV^ material.[7] Mixed Ru/Ir pyrochlores have to our knowledge not been previously reported.

Fine powders of composition (Na_0.33_Ce_0.67_)_2_(Ir_1−*x*_Ru_*x*_)_2_O_7_ were formed directly from hydrothermal reaction of metal chlorides in aqueous NaOH solutions containing a peroxide as oxidant. Figure 1 shows a typical powder XRD pattern, which can be indexed as a phase-pure, face-centered cubic pyrochlore.

**Figure 1:**
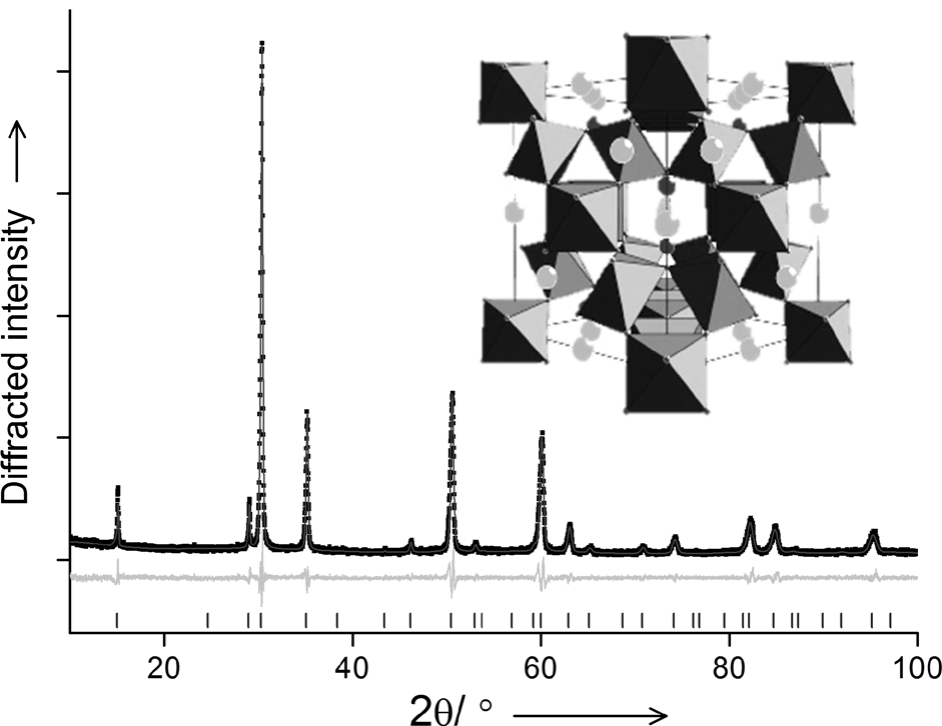
Refined powder X-ray diffraction pattern (*λ*=1.5406 Å) of (Na_0.33_Ce_0.67_)_2_(Ir_0.5_Ru_0.5_)_2_O_7_. The line is the fitted profile, black points the measured data, the pale line the difference and the ticks the positions of allowed Bragg reflections (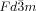, *a*=10.2242(3) Å). The inset is a view of the A_2_B_2_O_6_O′ pyrochlore (B-site octahedra, light grey A-site and dark grey O′).

The refined lattice parameter as a function of Ru/Ir ratio shows little variation, consistent with the almost identical six-coordinate radii of Ru^4+^ and Ir^4+^.[8] The pure iridium material shows lower crystallinity than the other materials in the series (see the Supporting Information, SI), but analysis of nitrogen adsorption isotherms (BET method) gave surface areas of typically 60–80 m^2^ g^−1^ for all samples, with no dependence on composition. The powder diffraction profile is broadened by small particle size, consistent with transmission electron microscopy (TEM) observations (Figure 2), but importantly energy dispersive X-ray analysis (EDXA) line-scans performed on numerous particles in the TEM show that Ru and Ir are uniformly distributed in the mixed-metal pyrochlores (Figure 2 c and d).

**Figure 2:**
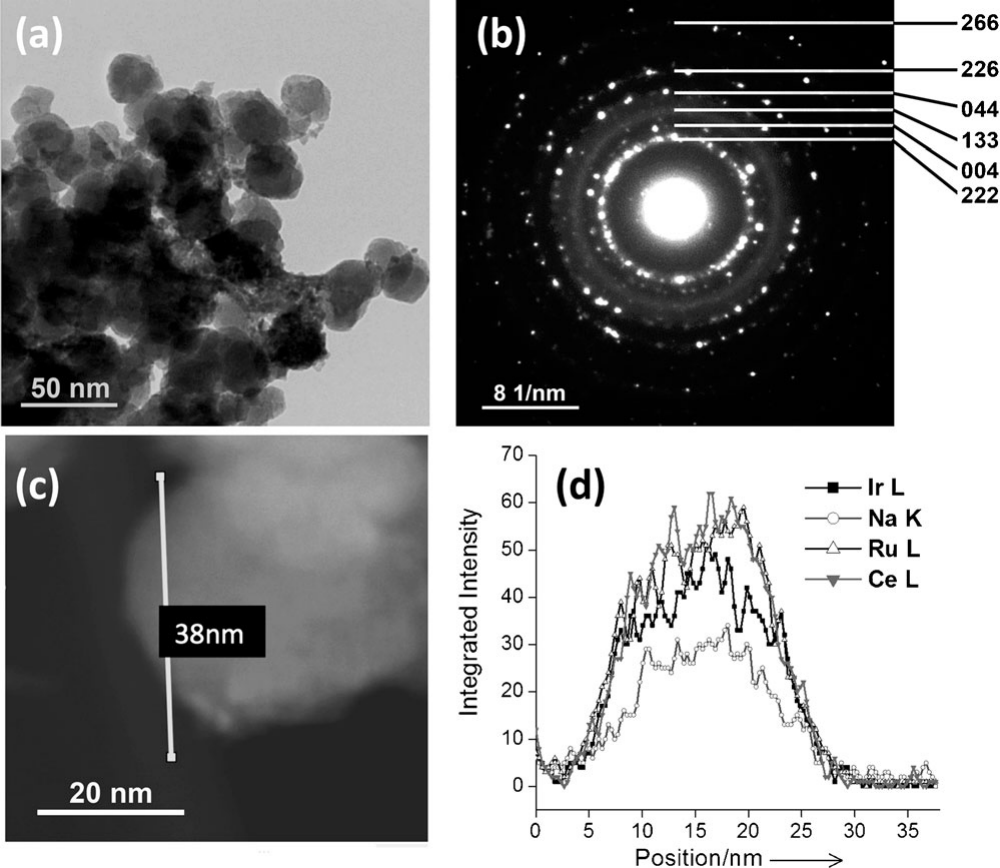
TEM analysis of (Na_0.33_Ce_0.67_)_2_(Ir_0.5_Ru_0.5_)_2_O_7_ nanoparticles; a) image of a typical region of specimen; b) SAED of agglomerated nanocrystals in (a) indexed using the F-cubic pyrochlore unit cell from XRD; c) representative single particle with the line profile region for the linear analysis in (d) indicated, and d) results of a typical EDXA line-scan analysis of particle in (c). Further EDXA lines scans are presented in the SI.

Carbon paper (Toray) backed electrodes were prepared using Nafion-based inks of the pyrochlore catalyst materials. Linear sweep voltammograms (Figure 3 a) of the electrodes in 0.5 m H_2_SO_4_ shows that for all the pyrochlore materials the onset of oxygen evolution occurs at ≈1.4 V vs. RHE, which is similar to the reference (Ru_0.9_Ir_0.1_)O_2_ and to other RuO_2_-based rutile materials described in the literature.[5] The onset was lowest for the (Na_0.33_Ce_0.67_)_2_Ru_2_O_7_ pyrochlore (1.35 V) and slightly higher for those materials that contained iridium (1.44 V). Measurement of the O_2_ (*m*=32) mass spectrometry signal (Figure 3 b), collected simultaneously with the voltammograms, shows that the oxygen evolution mass activity decreases with decreasing Ru content, whereas the carbon corrosion, measured as CO_2_ detected, is low (*m*=44; Figure 3 c). Analysis of impedance data (see Figure S2 and Table S2) shows that the oxygen evolution reaction proceeded with the lowest resistance for (Na_0.33_Ce_0.67_)_2_(Ru_0.5_Ir_0.5_)_2_O_7_ and that the highest resistance was observed for (Na_0.33_Ce_0.67_)_2_Ru_2_O_7_. Our pyrochlores show superior activity per gram Ru/Ir, when compared with a commercially available sample of (Ru_0.9_Ir_0.1_)O_2_, a material typical of those usually used for acid OER (see Table S2). A common way of quantifying the activity of such materials is to determine Tafel slopes;[5] we find that the pyrochlores give similar values to those reported for (Ru,Ir)O_2_ in the literature, illustrating the comparable electrocatalytic activity of the new materials and suggesting similar surface reactivity mechanism (Table S3).

**Figure 3:**
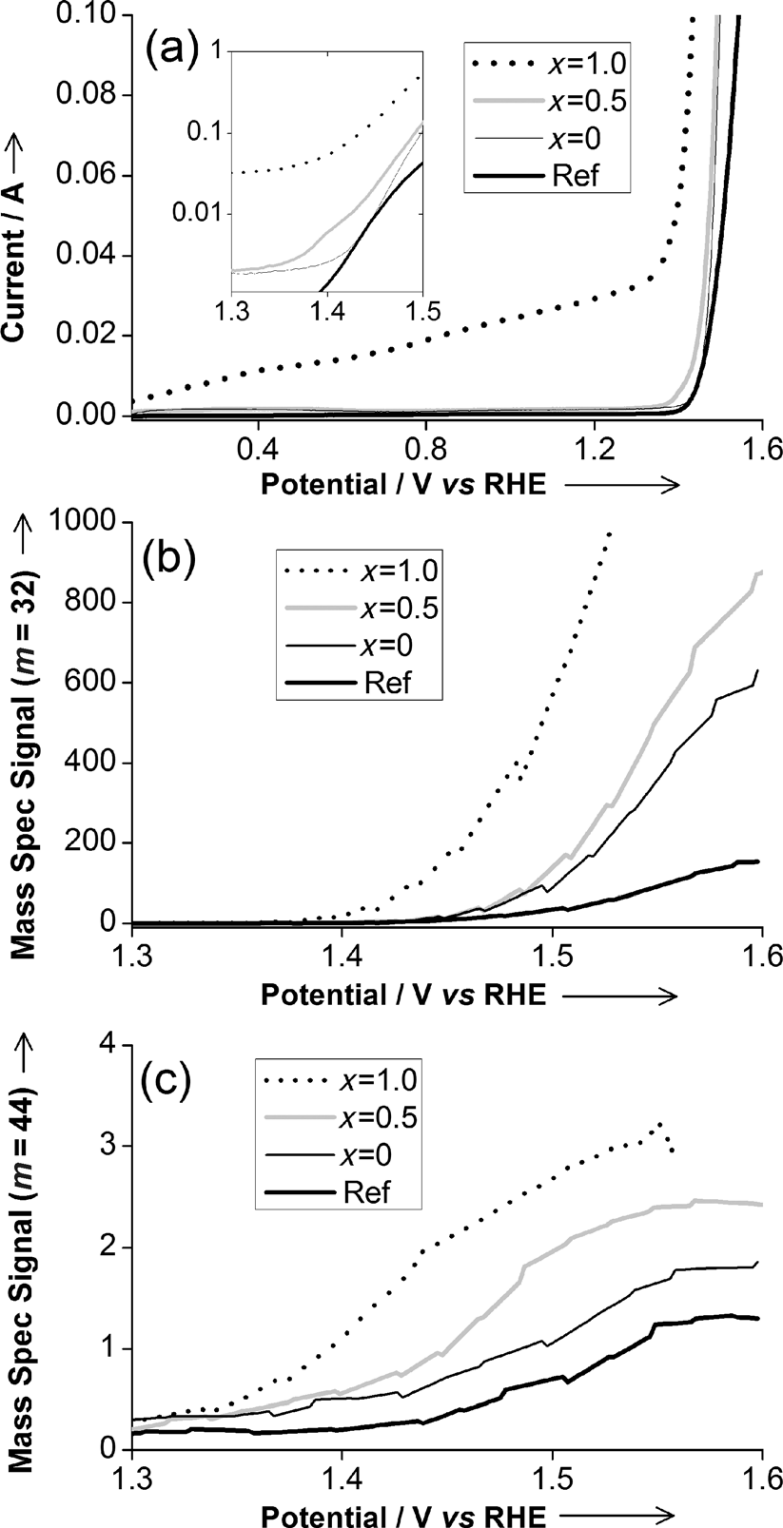
Differential electrochemical mass spectroscopy of (Na_0.33_Ce_0.67_)_2_(Ir_1−*x*_Ru_*x*_)_2_O_7_ pyrochlores and reference Ru-Ir rutile oxide (Ru_0.9_Ir_0.1_)O_2_ (Ref) during 10 mV s^−1^ potential sweep showing: a) current response, b) oxygen evolution, c) CO_2_ evolution (competing carbon corrosion).

With our new family of pyrochlores in hand we have used in situ X-ray absorption near edge structure (XANES) spectroscopy to examine changes in the local atomic environment of electrodes under potentiostatic control in 0.5 m aqueous H_2_SO_4_ electrolyte. Using an especially designed cell (SI) on B18 of the Diamond Light Source (UK) we were able to achieve OER conditions in acidic solution whilst recording the fluorescence XANES signal from the surface of the electrode. Previously reported in situ electrochemical studies of ruthenium and iridium oxide films did not reach OER conditions.[9] Initially we recorded ex situ Ru K edge and Ir L_III_ edge XANES of the pyrochlores and suitable reference materials containing oxygen-coordinated Ru and Ir in a range of oxidation states (Figure 4). These results confirm that the pyrochlores all contain both Ru and Ir in the +4 oxidation state. Once fabricated into electrode coatings and upon application of potential, systematic and reproducible shifts in the XANES signals are seen, which can be quantified from calibrant reference materials (SI). Over the duration of each in situ experiment, typically 2–3 h, there is no reduction in intensity of the XANES signal from either of the metals: this shows the robustness of the electrodes, with negligible loss of ruthenium or iridium into solution.

**Figure 4:**
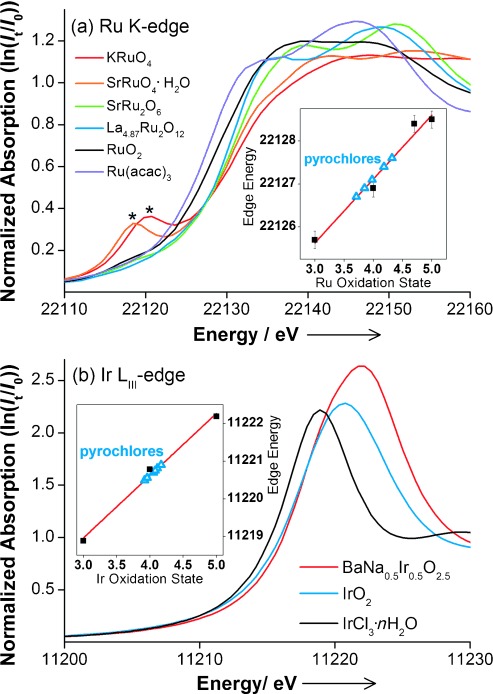
XANES spectra at a) Ru K edge [Ru^(+3)^(acetonylacetonate)_3_, Ru^(+4)^O_2_, La_4.87_Ru^(+4.70)^_2_O_12_, SrRu^(+5)^_2_O_6_, SrRu^(+6)^O_4_⋅H_2_O, and KRu^(+7)^O_4_] and b) Ir L_III_ edge (Ir^(+3)^Cl_3_⋅*n* H_2_O, Ir^(+4)^O_2_, and BaNa_0.5_Ir^(+5)^_0.5_O_2.5_). In (a) the 1s–4d transition of KRuO_4_ and SrRuO_4_⋅H_2_O is indicated by *. Insets show graphs of edge position versus oxidation state with the blue triangles for the pyrochlores giving the average oxidation state of +4.00 and +4.05 for Ru and Ir, respectively.

Figure 5 shows the changes in oxidation state versus potential applied determined from analysis of the in situ XANES data. It can be clearly seen that the onset of OER coincides with an oxidation of Ru or Ir, and both metals in the mixed materials. An important observation from the XANES experiments is that the response detected at each metal edge in the XANES experiment shows a distinct dependence on composition; this confirms that the materials are genuine mixed-metal compositions rather than physical mixtures of the two pure B-site metal end members (in which case we would expect identical results at each edge studied whatever the composition of the material). This corroborates the TEM results presented above regarding atomic-level homogeneity of the composition.

**Figure 5:**
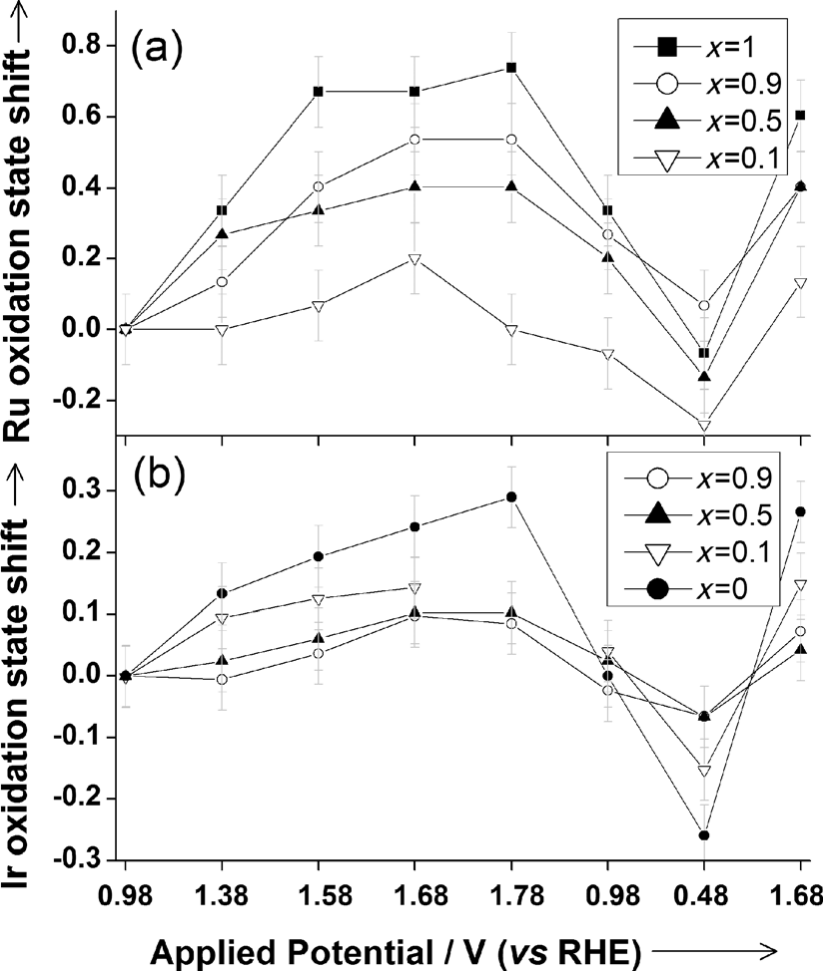
Plots of shifts in oxidation states of a) Ru and b) Ir derived from analysis of the in situ XANES spectra measured during electrochemistry of (Na_0.33_Ce_0.67_)_2_(Ir_1−*x*_Ru_*x*_)_2_O_7_ materials. Note that in (b) one data point is missing for the *x*=0.1 material due to interference from evolved oxygen bubbles.

Importantly, we see no evidence for the formation of tetrahedral Ru or Ir species in oxidation state higher than +5: indeed, the XANES signal from the tetrahedral [RuO_4_]^2−^ and [RuO_4_]^−^ anions, in addition to showing a distinctive shift to higher energies, also display a characteristic pre-edge 1s–4d transition (see Figure 4 a), which is never seen in our in situ data (SI). This would then suggest that at OER conditions a significant proportion of the Ru and/or Ir is oxidized from the +4 to the +5 oxidation state: because the XANES experiments use high-energy incident X-rays we are observing an average of all the Ru and Ir in each electrocatalyst layer. Upon reverse of the applied potential the metals are then reduced. The response in XANES edge shifts detected from the reference (Ru_0.9_Ir_0.1_)O_2_ material are much smaller compared to the pyrochlore materials (SI).

There has been much speculation as to the mechanism of action of ruthenium- and iridium-based oxides as electrocatalysts, with no real consensus reached in the literature.[3], [9] For example, some consider that a link between electrochemical activity and band structure of the solid exists,[10] or that the metal–oxygen binding energy plays a role in determining activity[11] and for mixed-metal oxides, the presence of oxygen vacancies may be important.[12] Ruthenate and iridate pyrochlore oxides have individually been studied for their activity in electrocatalysis, including the materials Bi_2_Ru_2_O_7−*δ*_, Bi_2_Ir_2_O_7_, and Pb_2_(Pb,M)_2_O_7−*δ*_ (M=Ru or Ir).[13], [14] The most detailed model for the electrocatalysis mechanism of such materials was the one proposed by Goodenough and co-workers.[12] They suggested that for oxide Pb_2_(Pb,Ir)_2_O_7−*δ*_ the displacement of surface hydroxide ions, linked to Pb, by superoxide anions maintains the octahedral coordination, but allows oxygen evolution by the Ir^4+^/Ir^5+^ couple through exchange of surface “O^−^” species. Recent work on iridium oxide films has used X-ray spectroscopy to examine local structure under moderate electrochemical potential and revealed the possibility of multiple Ir sites contributing to redox activity[15] and surface reactivity,[16] whereas for IrO_*x*_ dispersed in solution the oxidation of Ir to the +7 state has been proposed in water oxidation under basic conditions from conventional electrochemical measurements.[17] Our new in situ data are therefore more consistent with the Ir^4+^/Ir^5+^ model of Goodenough and co-workers, rather than with models that involve the oxidation of the precious metals to higher oxidation states than +5. In fact, our in situ study is the first that has attempted to observe directly the oxidation states of Ru and Ir during electrocatalysis under OER conditions: previous mechanistic studies of ruthenium and iridium oxides have inferred changes in oxidation state indirectly from electrochemical data,[3] and earlier in situ XAFS studies used lower applied potentials.[9], [15]

Our results reveal that in mixed ruthenium–iridium oxides both metals can contribute to the electrocatalytic activity and, indeed, show a cooperative effect that is composition-dependent. By this observation we have confirmed directly that the pyrochlore materials are atomically well-mixed phases and offer greater activity than a commercially available benchmark material. We have also provided direct evidence that the addition of Ir to the pure ruthenium pyrochlore tempers the activity of Ru, consistent with earlier results from (Ru,Ir)O_2_ materials.[5] Further mechanistic insight must come from the consideration of surface effects, to which the XAFS method (using high-energy incident X-rays) is rather insensitive. In terms of practical application in real devices, the long-term stability must also be determined, but given the wide substitutional chemistry possible for pyrochlore oxides,[18] it is anticipated that their electrochemical activity may be optimized for electrocatalysis applications by judicious doping with other metals.

## Experimental Section

Samples were prepared using hydrothermal synthesis within Teflon-lined stainless-steel autoclaves of internal volume ≈20 mL. For ruthenium-containing materials (Na_0.33_Ce_0.67_)_2_(Ir_1−*x*_Ru_*x*_)_2_O_7_ materials (*x*>0), the salts CeCl_3_⋅7 H_2_O, RuCl_3_⋅*x* H_2_O, and IrCl_3_⋅*y* H_2_O in the required molar ratio based on 0.19 g of the cerium salt, were stirred in 4 mL 5 m NaOH solution for 30 min. (The hydration value (*x* and *y*) of the ruthenium and iridium salts was determined by thermogravimetric analysis to allow accurate weighing.) Then 4 mL of H_2_O_2_ (30 % w/w in H_2_O) was added dropwise with continuous stirring (**CAUTION**: *the hydrogen peroxide can react violently with the solution of the metal salts*) before the mixture was sealed in the autoclave and transferred to a preheated fan oven where it was held at 225 °C for 5 days. For (Na_0.33_Ce_0.67_)_2_Ir_2_O_7_ the hydrogen peroxide was replaced by an equivalent amount of solid Na_2_O_2_ and the synthesis was performed at 240 °C for 5 days. All reactions yielded black, solid products, which were each recovered by suction filtration, washed with large amounts of distilled water and dried at 70 °C overnight in air. Details of the results of sample analysis and the in situ XANES experiments are described in the Supporting Information.
