# Supplementary material for: Water-Splitting Electrocatalysis in Acid Conditions Using Ruthenate-Iridate Pyrochlores
Source: Angew Chem Int Ed Engl. 2014 Sep 4;53(41):10960–4. doi: 10.1002/anie.201406668 (PMC4497602; doi:10.1002/anie.201406668)
Supplement: Supplementary file 1 [file anie0053-10960-sd1.pdf]

Angewandte Chemie
Eine Zeitschrift der Gesellschaft Deutscher Chemiker

Supporting Information
© Wiley-VCH 2014
69451 Weinheim, Germany

Water-Splitting Electrocatalysis in Acid Conditions Using Ruthenate-Iridate Pyrochlores**

Kripasindhu Sardar, Enrico Petrucco, Craig I. Hiley, Jonathan D. B. Sharman, Peter P. Wells, Andrea E. Russell, Reza J. Kashtiban, Jeremy Sloan, and Richard I. Walton**

anie_201406668_sm_miscellaneous_information.pdf

Contents

	Page
**S1: Sample analysis**	2
**S2: Electrochemistry**	4
**S3: XANES experiment and calibration**	6
**S4: *In situ* XANES**	8

S1: Sample analysis

Powder XRD was performed using a Panalytical X'Pert Pro MRD quipped with a focussing Johanson monochromator on the incident beam optics to give high-resolution pure Cu Kα1 radiation. Patterns were analysed using the Pawley Method within the TOPAS software (TOPAS-Academic V5, Coelho Software, Brisbane, Australia). Electron microscopy and EDXA were performed using a JEOL ARM200F double aberration corrected instrument equipped with an Oxford Instrument X-Mas T spectrometer, operating at 80 kV on specimens that were dispersed by ultrasound in ethanol and dropped onto 3 mm lacey carbon grids supplied by Agar. EDXA, with typically 10 regions of sample, analysed gave good agreement with the expected chemical composition; see Table S1.

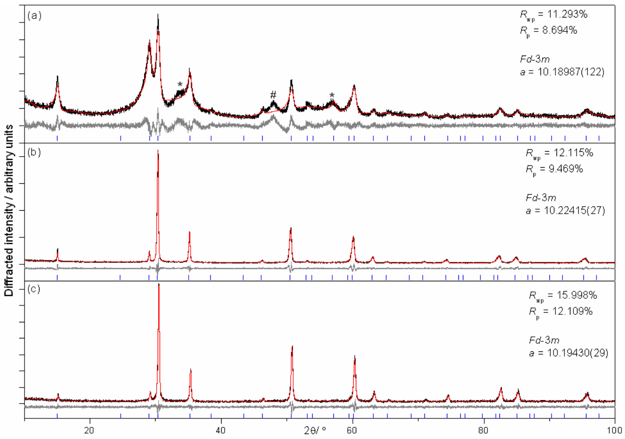

Figure S1: Refined powder XRD patterns for (a) $(Na_{0.33}Ce_{0.67})_2(Ir)_2O_7$ (b) $(Na_{0.33}Ce_{0.67})_2(Ir_{0.5}Ru_{0.5})_2O_7$ and (c) $(Na_{0.33}Ce_{0.67})_2(Ru)_2O_7$. In (a) * and # indicate small amounts of IrO_2 and CeO_2 impurity, respectively.

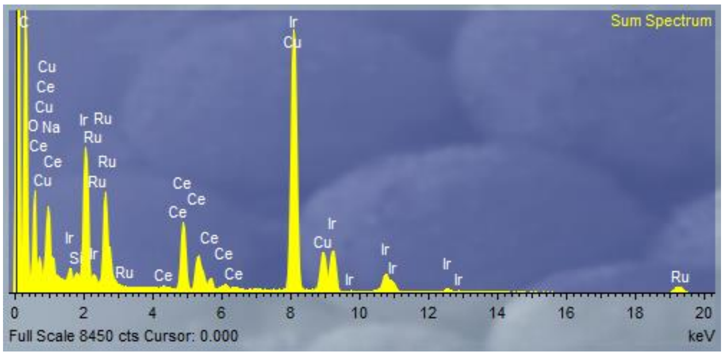

Figure S2: Typical EDXA Spectrum of $(Na_{0.33}Ce_{0.67})_2(Ir_{0.5}Ru_{0.5})_2O_7$ measured by TEM.

Table S1: EDXA analysis results: relative composition given with expected values in parentheses.

**Material**	**Na**	**Ce**	**Ru**	**Ir**
$(Na_{0.33}Ce_{0.67})_2Ru_2O_7$	0.32(0.33)	0.82(0.67)	1(1)	-
$(Na_{0.33}Ce_{0.67})_2(Ir_{0.5}Ru_{0.5})_2O_7$	0.56(0.67)	1.17(1.33)	1(1)	0.94(1)
$(Na_{0.33}Ce_{0.67})_2Ir_2O_7$	0.29(0.33)	0.61(0.67)	-	1(1)

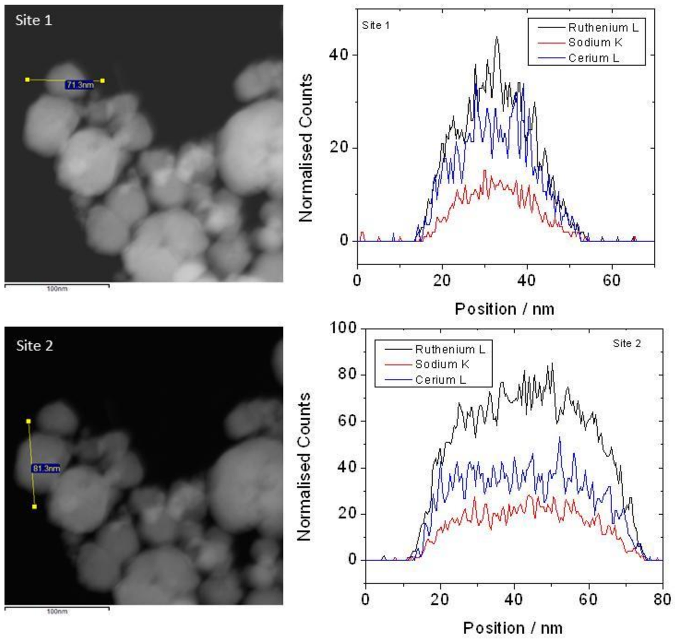

Figure S3: HAADF-STEM images of $(Na_{0.33}Ce_{0.67})_2Ru_2O_7$ with EDXA linescan analysis

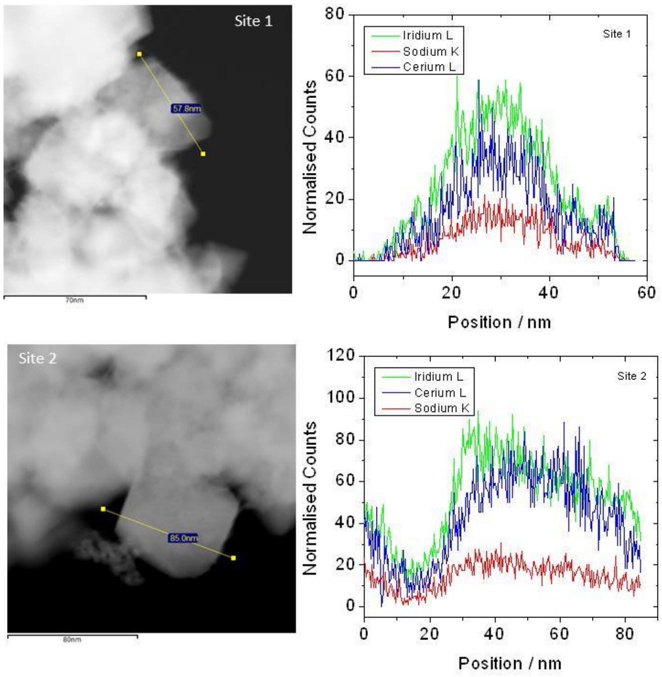

Figure S4: HAADF-STEM images of $(Na_{0.33}Ce_{0.67})_2Ir_2O_7$ with EDXA linescan analysis

TGA/DSC (using a Mettler-Toledo TGA/DSC1 instrument) were recorded under a flow of air with a 10 °C min^{-1} heating rate. All show ~ 5 % surface water/hydroxide mass loss to ~ 600 ° C. The Ir-containing materials additionally show mass loss at >900 °C which corresponds to reduction to Ir metal; this generally increases with increasing Ir content in the series of materials (athough the mass loss is not always complete at 1000 °C.

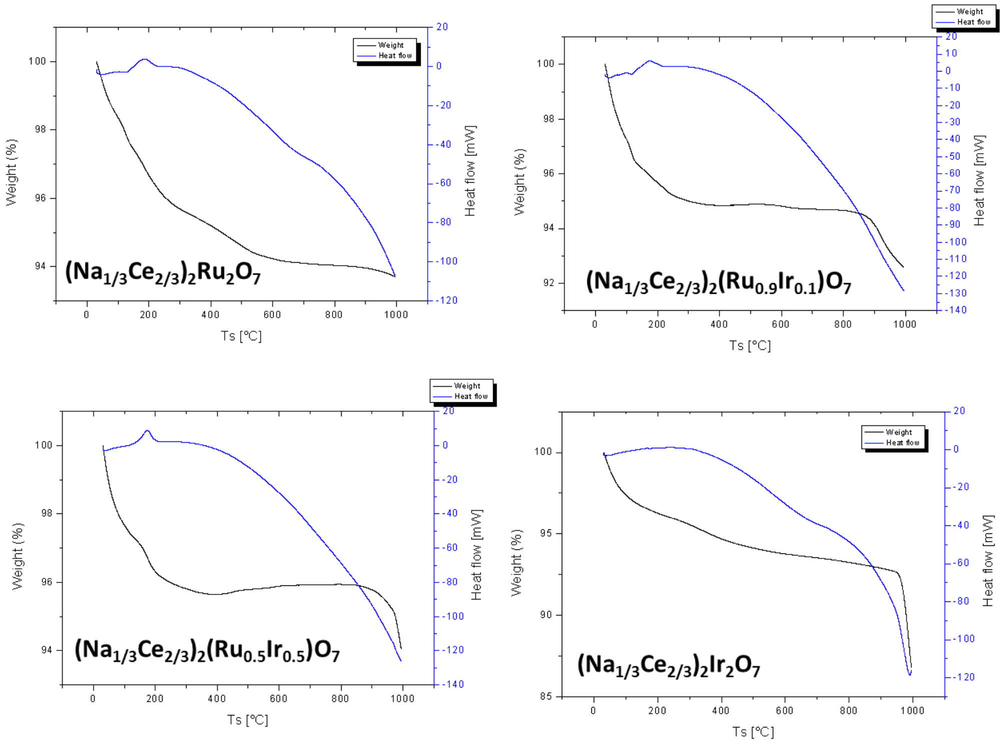

Figure S5: TGA-DSC of $(Na_{0.33}Ce_{0.67})_2(Ir_{1-x}Ru_x)_2O_7$ materials

S2: Electrochemistry

The catalyst powders were mixed into inks using Nafion as binder and applied onto microporous layered Toray TGP-H-060 to make electrodes. Two sets of electrodes were prepared: the PGM metal content for the first set targeted a loading (0.5 mg cm^{-2} PGM) designed to collect satisfactory XANES data while the PGM metal content target was 0.1 mg cm^{-2} for the second set, which were used for electrochemical characterisation at Johnson Matthey Technology Centre. Electrodes were assembled into a 6 cm² active area membrane electrode assembly (MEA) using N112-type Nafion and 0.4 mg cm^{-2} Pt (HiSPEC9100) as the counter electrode. The cell was held at 80 °C with fully humidified reactants. Testing was performed with 200 sccm N_2 flow at the working electrode and 167 sccm H_2 flow at the counter/reference electrode. Current and potential were controlled using an Eco Chemie Autolab PGStat30 operated with Nova 1.6 software. The potential was cycled between 10-1600 mV at 10mV s^{-1}. The oxygen evolution onset was defined at 1mA cm^{-2} above the background current. The mass activity was determined at 1.5 V vs RHE.

Polarisation curves were obtained by logarithmically spaced galvanostatic steps with electrochemical impedance spectroscopy at each step. The EIS curves were fitted using a resistor, inductor, and Cole-element in series as the equivalent circuit using ZView. Exhaust gas was fed to a Spectra mass spectrometer and continuously monitored throughout the entire test protocol for oxygen evolution and carbon corrosion products from competing side reactions. Electrochemical work at Diamond Light Source used a Hg/Hg_2SO_4 (MMS) reference electrode in 0.5M H_2SO_4 which was calibrated against a hydrogen reference electrode off site at −0.68V *vs* RHE such that 1.00 V *vs* MMS signal was equivalent to 1.68 V *vs* RHE. Table S2 shows the mass activity and onset for oxygen evolution data collected during voltammetry. Also included in the table are the fit parameters found for data collected at 10 mA cm^{-2} during polarisation. The parameters indicate DC resistance, R_0, charge transfer resistance, R_{ct}, capacitive time constant, τ, and phase, α.

Table S2: Electrochemical Characterisation of $(Na_{0.33}Ce_{0.67})_2(Ir_{1-x}Ru_x)_2O_7$ materials (Ref is a standard $(Ru_{0.9}Ir_{0.1})O_2$ rutile oxide (supplied by Johnson Matthey); PGM = platinum group metal). See above text for definition of terms; χ^2 is a goodness of fit for the data fitting.

	**Mass Act$_{1.5V}$**	**Mole Act$_{1.5V}$**	**Onset**	R_0	R_{ct}	**Capacitive Time Constant**	**Phase**	χ^2	**Overpotential**
	A/ g PGM	A/ mmol PGM	V *vs* RHE	Ω cm	Ω cm	τ	α		V
x= 1.0	1359	137.4	1.348	0.125	4.56	0.042	0.811	0.0009	0.214
x= 0.5	238	34.9	1.411	0.115	2.48	0.074	0.801	0.0023	0.252
x= 0.0	189	36.3	1.441	0.119	2.56	0.060	0.806	0.0018	0.278
Ref	51	5.6	1.439	0.134	4.07	0.019	0.844	0.0023	0.335

Table S3: Tafel Slope analysis of pyrochlores compared to literature values for $(Ru_xIr_{1-x})O_2$ materials.

**Pyrochlore $(Na_{0.33}Ce_{0.67})_2(Ir_{1-x}Ru_x)_2O_7$**	**Tafel Slope / mV decade^{-1}**	**Rutile $(Ru_xIr_{1-x})O_2$[a]**	**Tafel Slope / mV decade^{-1}**
x= 1.0	48.6	x= 1.0	40.9
		x= 0.8	49.9
x= 0.5	67.3	x= 0.5	56.9
		x= 0.3	59.8
x= 0.0	85.6	x= 0.0	58.6

a: literature data taken from Kotz and Stucki, *Electrochemica Acta*, **1986**, *31*, 1311 for $(Ru_xIr_{1-x})O_2$ in 1 N H_2SO_4.

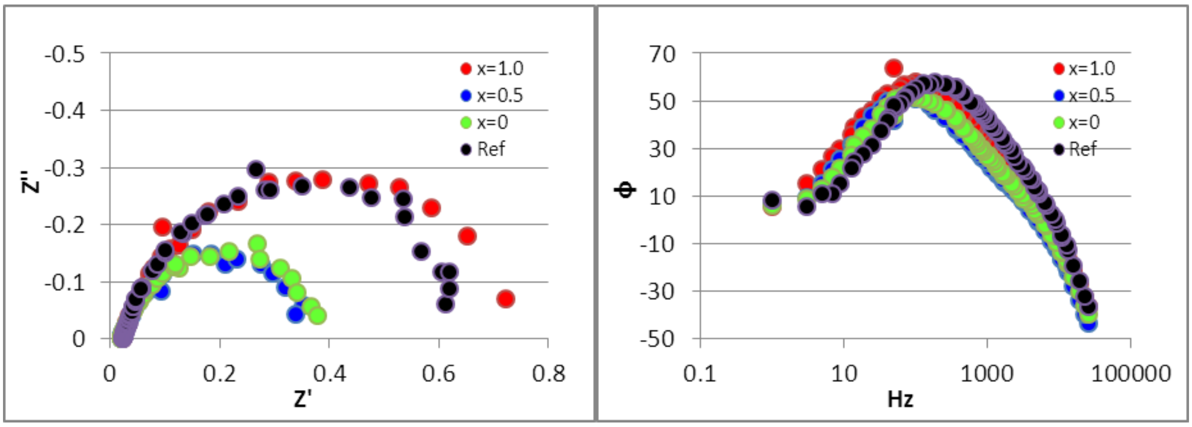

Figure S6: Electrochemical Impedance Spectroscopy at 10 mA cm^{-2} for electrode coatings of $(Na_{0.33}Ce_{0.67})_2(Ir_{1-x}Ru_x)_2O_7$ materials as electrode coatings compared to the standard (Ru,Ir)O_2 electrocatalyst (labelled Ref).

S3: XANES experiment and calibration

XANES experiments were performed using beamline B18 of the Diamond Light Source, UK (Dent, A. J.; Cibin, G.; Ramos, S.; Smith, A. D.; Scott, S. M.; Varandas, L.; Pearson, M. R.; Krumpa, N. A.; Jones, C. P.; Robbins, P. E. In *14th International Conference on X-Ray Absorption Fine Structure*; DiCicco, A., Filipponi, A., Eds.; Iop Publishing Ltd: Bristol, 2009; Vol. 190). This beamline provides X-ray energies in the range 2.05–35 keV using either a fixed-exit, double-crystal Si(111) monochromator, or a Si(311) monochromator, which provide an energy resolution of typically 2×10^4. The optics of the beamline include a Pt-coated collimating mirror and a toroidal focussing mirror before and after the monochromator, respectively. Under this configuration, the expected flux on the sample is of the order of 5×10^{11} photons s^{-1} and the size of the beam at that position is approximately 0.6 mm in the vertical direction by 1 mm in the horizontal one. XANES data were collected at the Ru K and Ir L_{III} edges in transmission mode for the reference materials with ion chambers before and behind the sample filled with appropriate mixtures of inert gases to optimise sensitivity. The spectra were measured with a step size equivalent to less than 0.5 eV.

The reference materials $Ru(acac)_3$, RuO_2, $KRuO_4$, $IrCl_3{\cdot}nH_2O$ and IrO_2, were used as supplied by Alfa-Aesar, with their identities confirmed by powder X-ray diffraction. $La_{4.87}Ru_2O_{12}$ was prepared according to the method of Khalifah *et al.* (*J. Solid State Chem.* **155** (2000) 189-197), $SrRu_2O_6$ from our own work (C.I. Hiley, *et al.*, *Angew. Chem., Int. Ed.* **53** (2014) 4423-4427), $SrRuO_4{\cdot}H_2O$ was prepared from a hydrothermal reaction between SrO_2 and $KRuO_4$ in 0.5M KOH at 140 ^{o}C for 24 hours, and $BaNa_{0.5}Ir_{0.5}O_{2.5}$ by the method of Jung *et al.* (*Mater. Res. Bull.* **30** (1995) 113-123). Small samples (~20 mg) of materials to be studied were ground finely with polyethylene powder (~

80 mg) under acetone and after evaporation of the solvent pressed into 13 mm diameter pellets of ~1 mm thickness under a pressure of 5 Tonnes. The precise dilution was chosen to optimised the edge step with minimal self absorption. Data were normalised using the program Athena (Ravel, B.; Newville, M. *J. Synchrot. Radiat.* **2005**, *12*, 537) with a linear pre-edge and polynomial post-edge background subtracted from the raw $\ln(I_t/I_0)$ data.

The Ru K-edge was defined as the energy of 50 % of the edge step. The calibration graph obtained from the reference materials (Figure 4a) gave the following relationship:

Ru O.S. = (edge – 22121.17)/1.49

This agrees with Planas *et al.* (*Inorg. Chem.* 2011, **50**, 11134–11142) who studied a variety of Ru materials using K-edge XANES. Using the tetrahedral reference compound to add to the oxidation state calibration graph is not possible since the pre-edge feature overlaps with the K-edge, making the same edge determination (as for the octahedral materials) impossible. Planas *et al.* similarly used reference materials containing octahedral Ru in oxidation states +2,+3 and +4 to determine a calibration graph, which could be extrapolated to higher oxidation states.

The Ir L_{III}-edge can most easily be defined as position of white line. This analysis agrees well with the results of Mugavero *et al.* (*Angew. Chem.* **48** (2009) 215-218), and this corresponds to the second minimum in the second derivative plot, used by Choy et al. (*J. Phys. Chem.*, **98** (1994) 98, 6258 and *J. Am. Chem. Soc.*, **117** (1995) 8557). The white line position was determined using a single Gaussian fit over the range 11210-11230 eV following subtraction of a linear background (extrapolation from first to last point over this range).

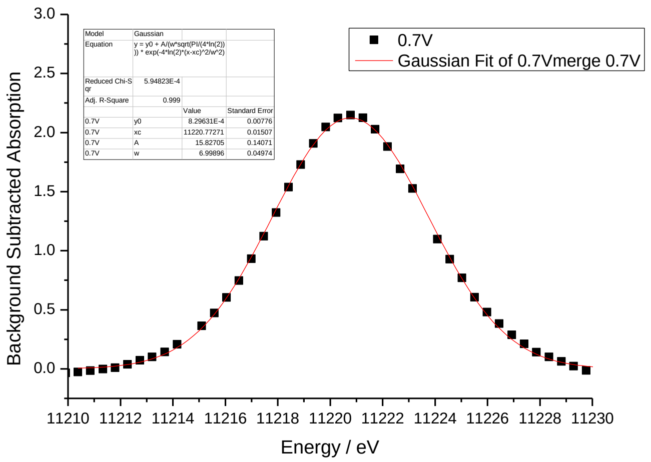

Figure S7: Example of Gaussian fit to Ir L_{III}-edge

The calibration graph obtained from the reference materials (Figure 4b) gave the following relationship:

Ir O.S. = (edge – 11214)/1.655

Table S4: Ru K-edge of Electrode Materials as Dry Powders

Material	Edge Position / eV	Ru Oxidation State
$(Na,Ce)_2Ru_2O_{7-\delta}$	22127.4	4.18
$(Na,Ce)_2(Ir_{0.1}Ru_{0.9})_2O_{7-\delta}$	22127.1	3.98
$(Na,Ce)_2(Ir_{0.5}Ru_{0.5})_2O_{7-\delta}$	22127.6	4.32
$(Na,Ce)_2(Ir_{0.7}Ru_{0.3})_2O_{7-\delta}$	22126.9	3.85
$(Na,Ce)_2(Ir_{0.8}Ru_{0.2})_2O_{7-\delta}$	22127.1	3.98
$(Na,Ce)_2(Ir_{0.9}Ru_{0.1})_2O_{7-\delta}$	22126.7	3.71

Table S5: Ir L_{III}-edge of Electrode Materials as Dry Powders

Material	Edge Position / eV	Ir Oxidation State
$(Na,Ce)_2(Ir_{0.1}Ru_{0.9})_2O_{7-\delta}$	11220.81	4.11
$(Na,Ce)_2(Ir_{0.5}Ru_{0.5})_2O_{7-\delta}$	11220.7	4.05
$(Na,Ce)_2(Ir_{0.7}Ru_{0.3})_2O_{7-\delta}$	11220.49	3.92
$(Na,Ce)_2(Ir_{0.8}Ru_{0.2})_2O_{7-\delta}$	11220.56	3.96
$(Na,Ce)_2(Ir_{0.9}Ru_{0.1})_2O_{7-\delta}$	11220.75	4.08
$(Na,Ce)_2Ir_2O_{7-\delta}$	11220.9	4.17

S4: *In situ* XANES

The cell design is shown below in Figure S8. This is a 30 cm^3 volume cell construction from glass with Perspex windows that uses a Pt counterelectrode and MMS reference electrode. The cell was filled with fresh 0.5 M H_2SO_4 with each electrode. The reference electrode was calibrated in 0.5M H_2SO_4 against a hydrogen reference electrode at –0.68V *vs* RHE.

The electrodes were prepared as described above. The catalysed electrode sections were boiled in type I purified water and then place under vacuum for a minimum of two hours in order to fill the porosity. The cell uncompensated resistance will have caused the actual potential to be lower than the measured potential. However, the current was low so any resistance should require only minimal potential correction.

The *in situ* electrochemistry measurements were carried out in fluorescence mode on B18 using a germanium 9-element detector with XPRESS data acquisition electronics. The Ru K-edge and Ir L_{III}-edge experiments were performed separately using different electrodes but made from pyrochlores from the same batch, since concentration of each of the metals to be studied had to be optimised separately for each edge. The measurements were made with circulating electrolyte and

the data collection was monitored continuously to ensure that bubble formation did not intefere with the data collection; if necessary the detector was repositioned during the measurement.

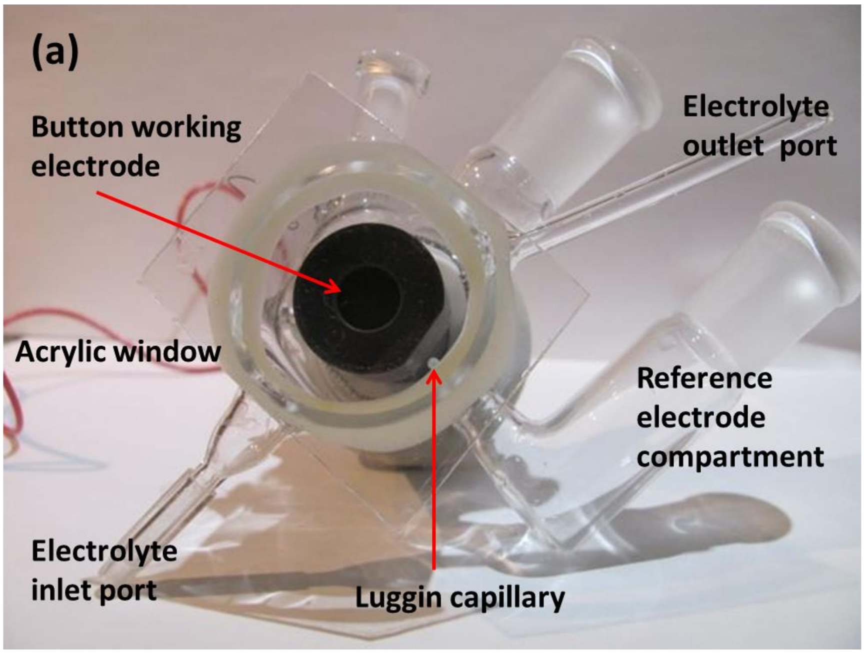

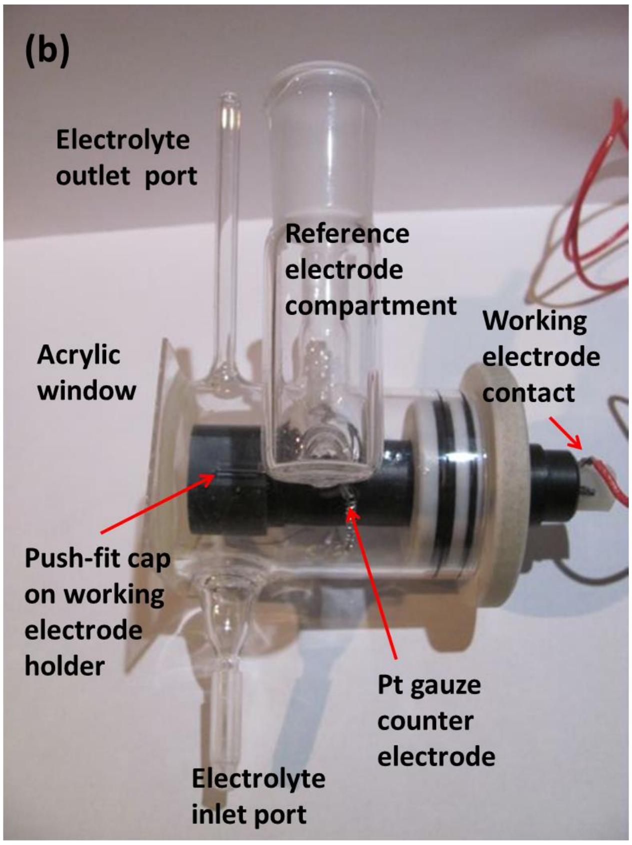

Figure S8: (a) View of the* in situ *XANES cell showing electrode presented to fluorescence detector (b) view sideways on showing working electrode contact.

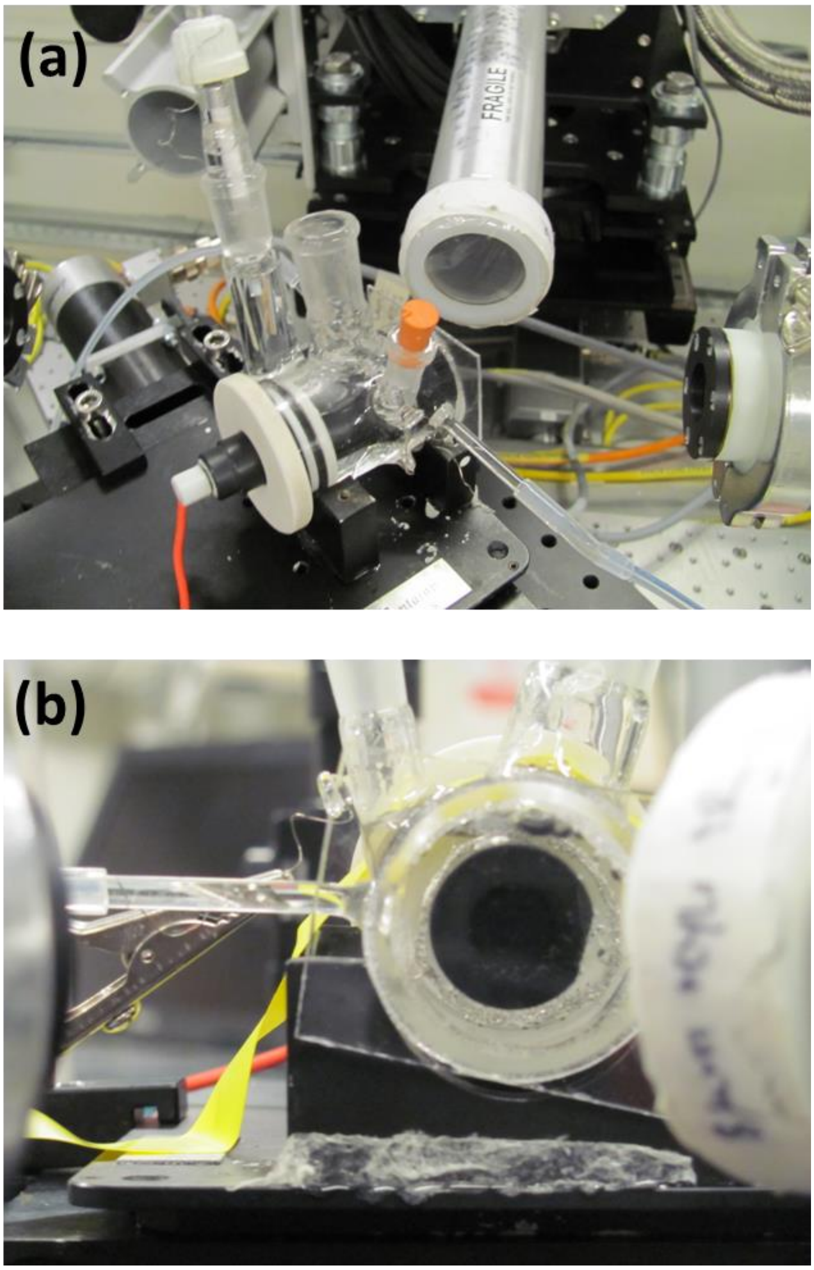

Figure S9: (a) photograph of the* in situ *XANES cell assembled and (b) in use on B18 at OER conditions.

Data were recorded at 0.3, 0.7, 0.9, 1.0, 1.1, 0.3 -0.2, 1.0 V *vs* MMS in sequence with 4 XAFS scans per voltage and sufficient post-edge background to allow normalisation to produce XANES spectra. The plots are expressed as potentials *vs* RHE (+0.68 V). In the plots below at the Ru K-edge, note the absence of any pre-edge feature, showing no evidence for 1*s*-4*d* transition (at 22120 eV for tetrahedral Ru(VII) or 22118 eV for tetrahedral Ru(VI)) in any of the spectra (see Figure 4, main text).

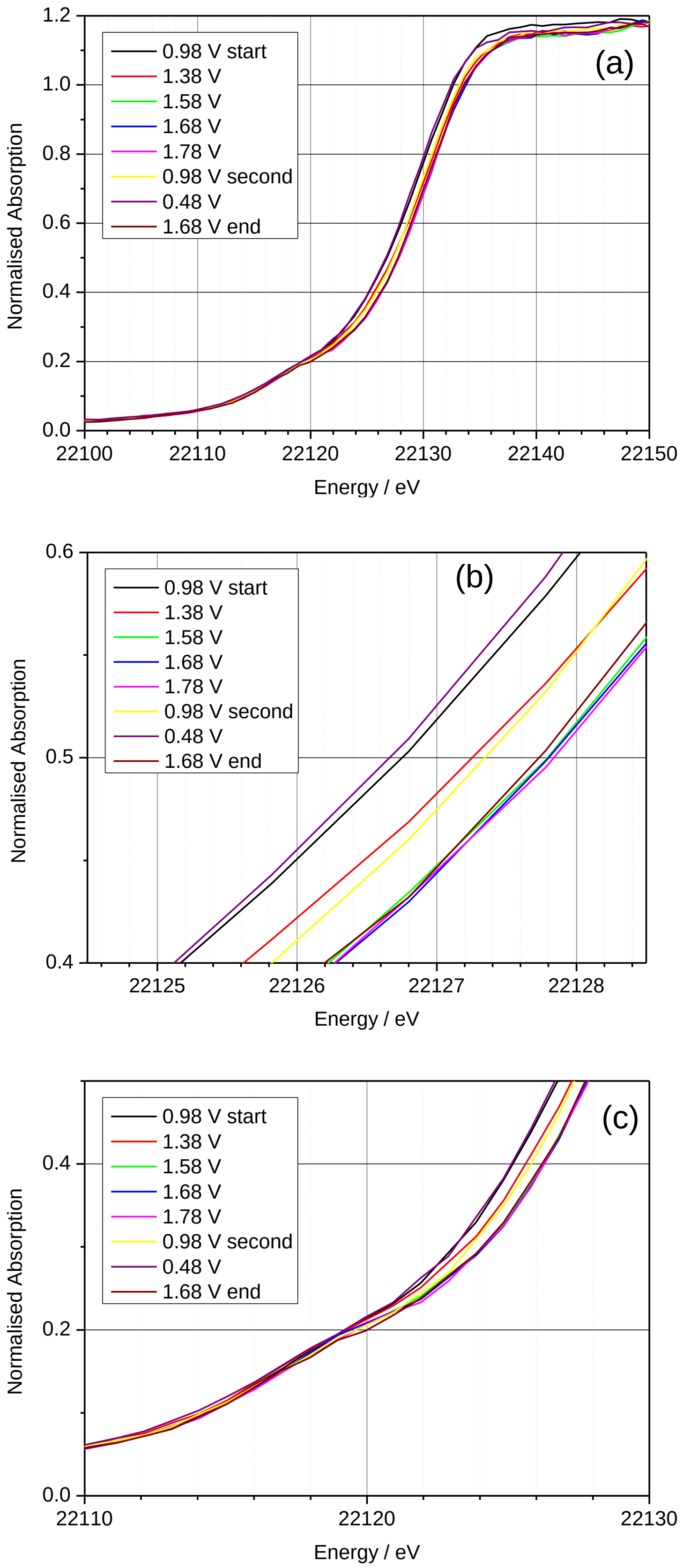

Figure S10: Ru K-edge XANES in situ with applied potential of $(Na_{0.33}Ce_{0.67})_2Ru_2O_7$ (a) XANES spectrum, (b) near-edge region and (c) pre-edge region.

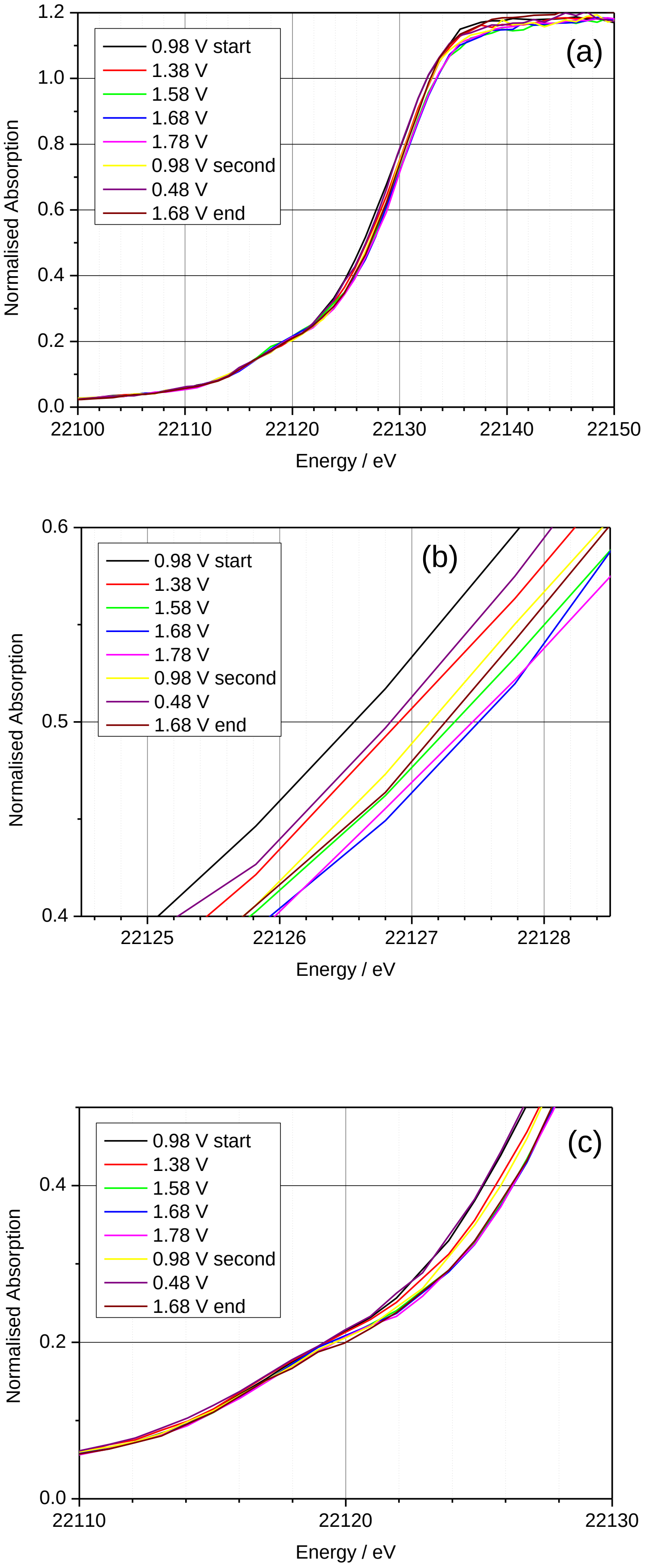

Figure S11: Ru K-edge XANES in situ with applied potential of $(Na_{0.33}Ce_{0.67})_2(Ir_{0.1}Ru_{0.9})_2O_7$ (a) XANES spectrum, (b) near-edge region and (c) pre-edge region.

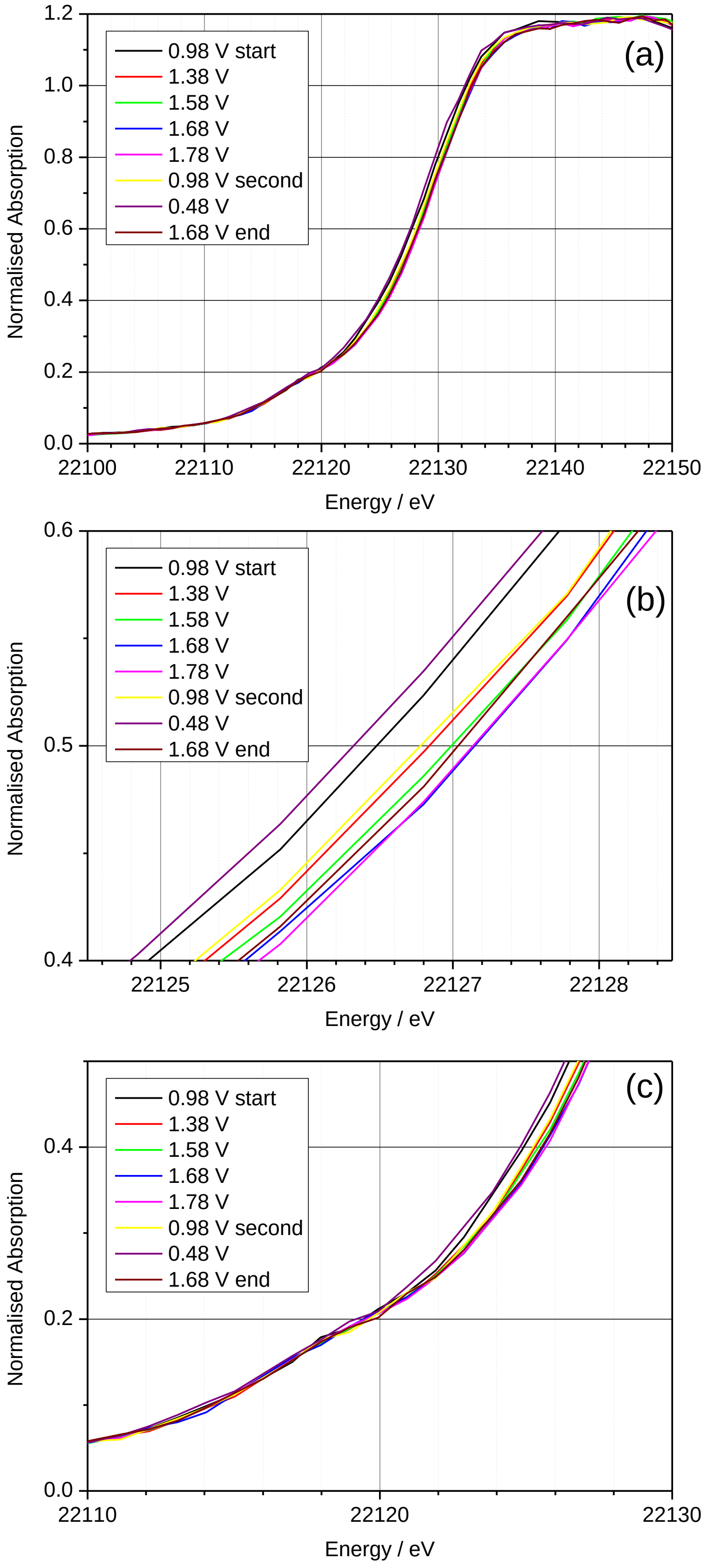

Figure S12: Ru K-edge XANES in situ with applied potential of $(Na_{0.33}Ce_{0.67})_2(Ir_{0.5}Ru_{0.5})_2O_7$ (a) XANES spectrum, (b) near-edge region and (c) pre-edge region.

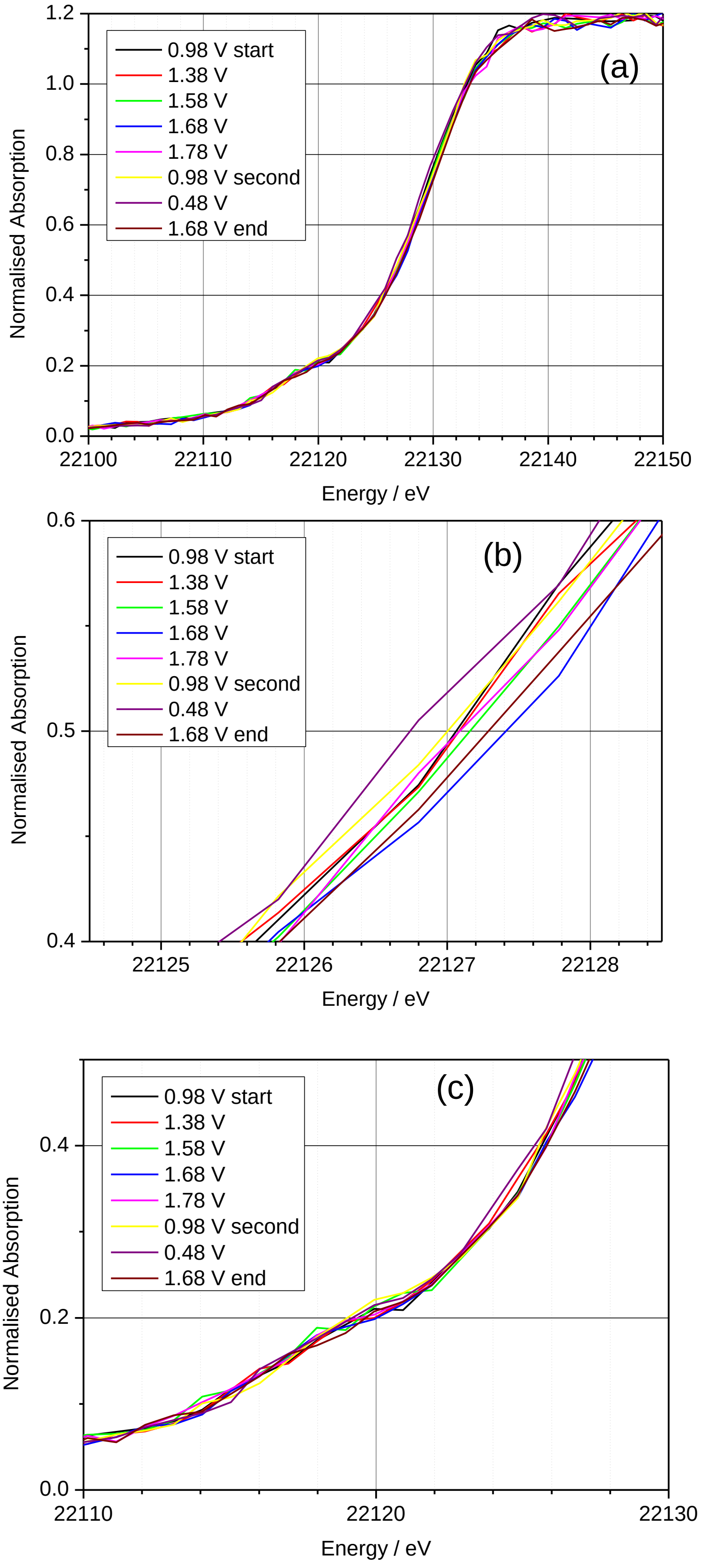

Figure S13: Ru K-edge XANES in situ with applied potential of $(Na_{0.33}Ce_{0.67})_2(Ir_{0.9}Ru_{0.1})_2O_7$ (a) XANES spectrum, (b) near-edge region and (c) pre-edge region.

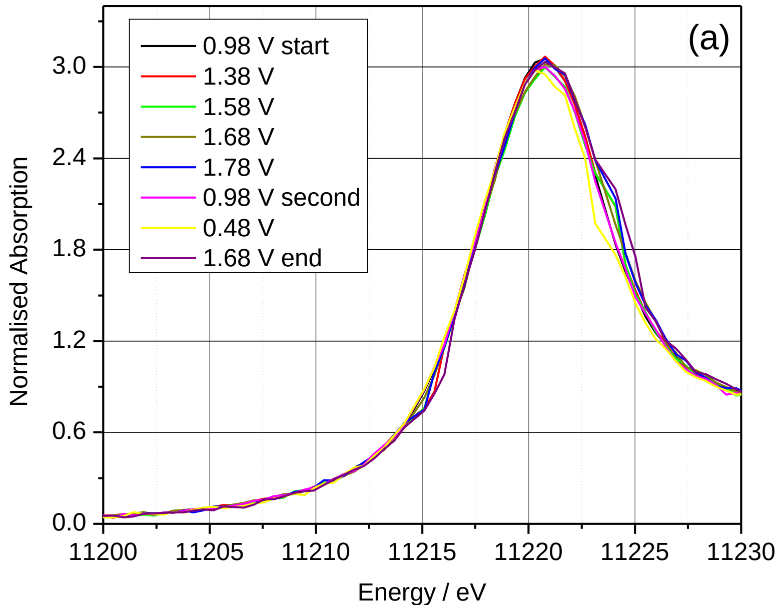

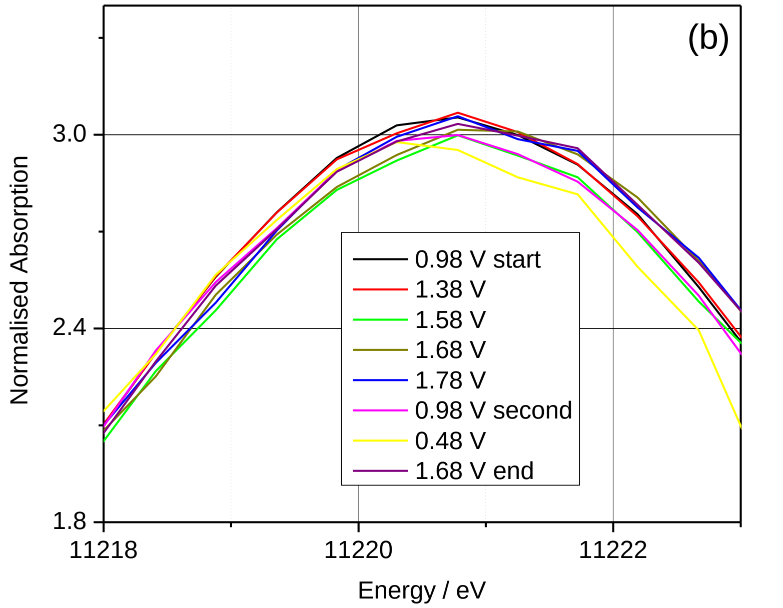

Figure S14: Ir L_{III}-edge XANES in situ with applied potential of $(Na_{0.33}Ce_{0.67})_2(Ir_{0.1}Ru_{0.9})_2O_7$ (a) XANES spectrum and (b) near-edge region.

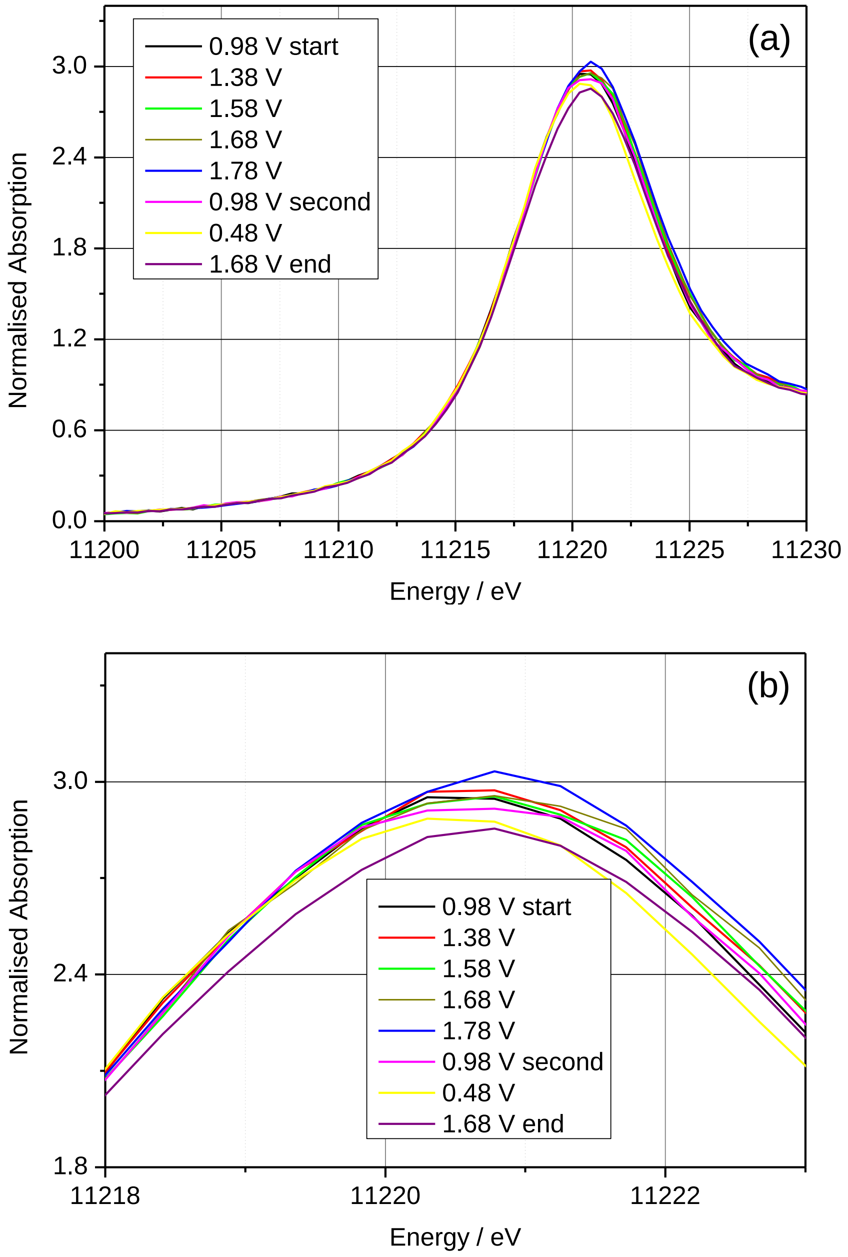

Figure S15: Ir L_{III}-edge XANES in situ with applied potential of $(Na_{0.33}Ce_{0.67})_2(Ir_{0.5}Ru_{0.5})_2O_7$ (a) XANES spectrum and (b) near-edge region.

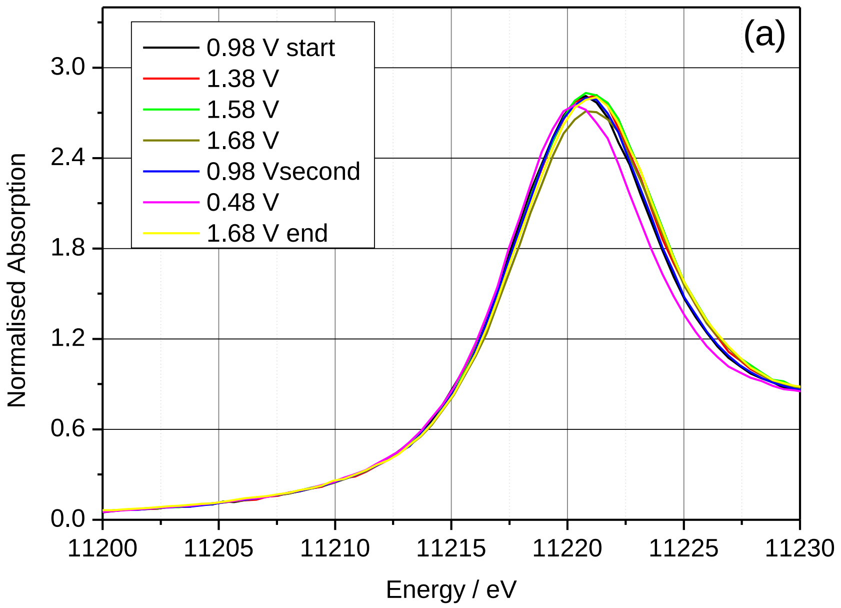

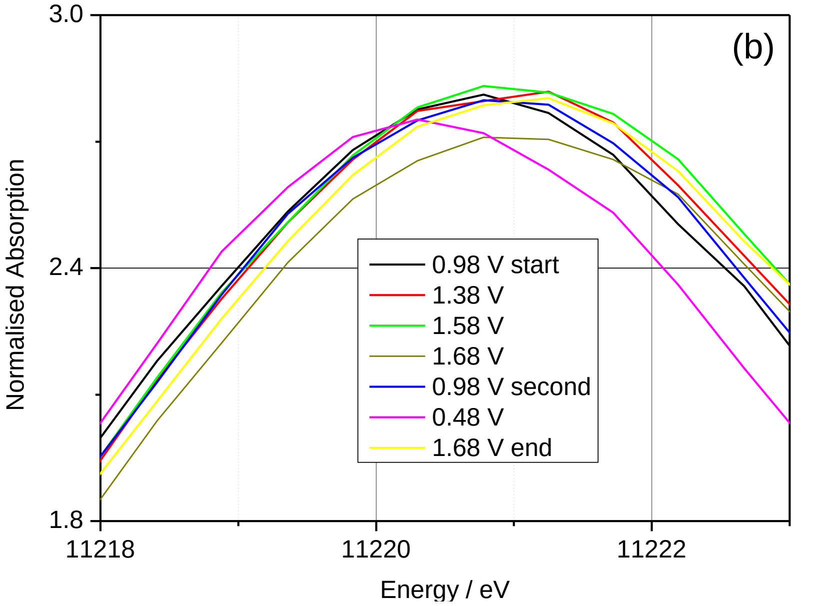

Figure S16: Ir L_{III}-edge XANES in situ with applied potential of $(Na_{0.33}Ce_{0.67})_2(Ir_{0.9}Ru_{0.1})_2O_7$ (a) XANES spectrum and (b) near-edge region.

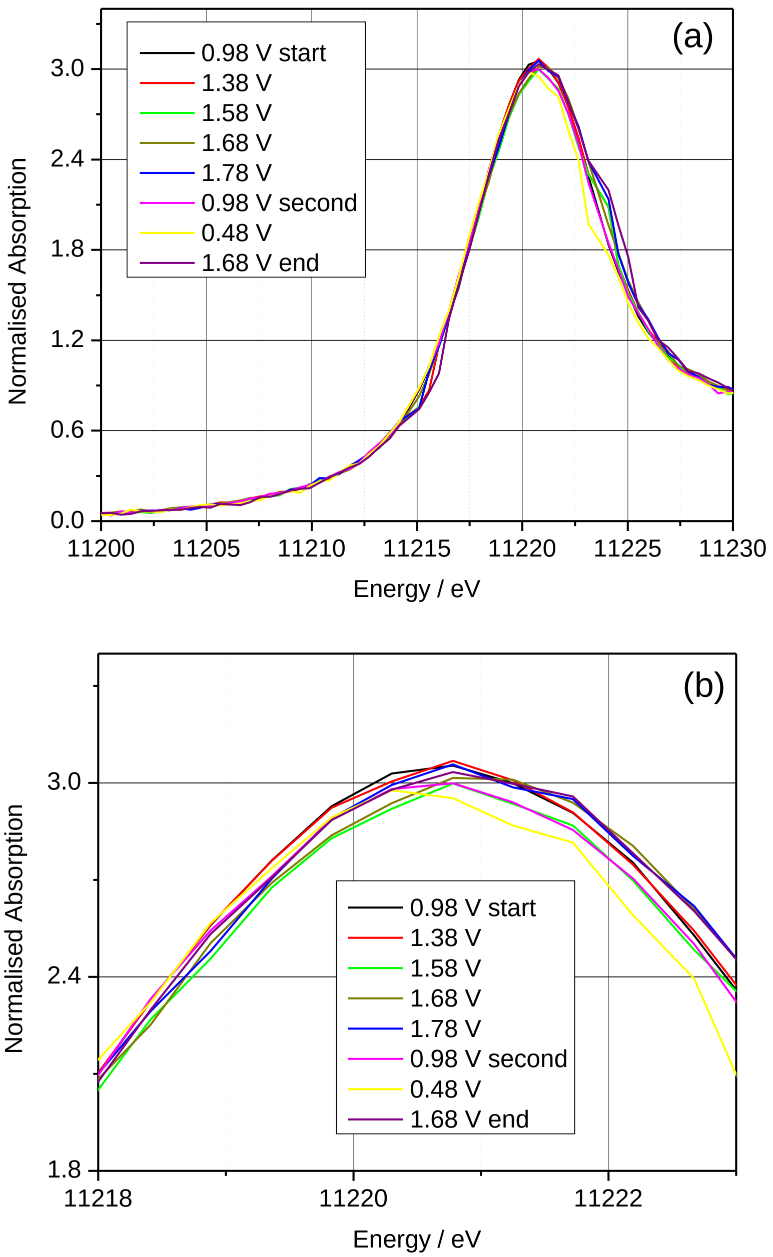

Figure S17: Ir L_{III}-edge XANES in situ with applied potential of $(Na_{0.33}Ce_{0.67})_2Ir_2O_7$ (a) XANES spectrum and (b) near-edge region.

Table S6: Analysis of in situ Ru K-edge XANES data (for fitting procedure see method described above for reference materials).

Material→	$(Na_{0.33}Ce_{0.67})_2Ru_2O_7$		$(Na_{0.33}Ce_{0.67})_2(Ir_{0.1}Ru_{0.9})_2O_7$		$(Na_{0.33}Ce_{0.67})_2(Ir_{0.5}Ru_{0.5})_2O_7$		$(Na_{0.33}Ce_{0.67})_2(Ir_{0.9}Ru_{0.1})_2O_7$	
Applied potential *vs* RHE	Edge Position	Ru Oxidation State	Edge Position	Ru Oxidation State	Edge Position	Ru Oxidation State	Edge Position	Ru Oxidation State
0.98 V	22126.8	3.8	22126.7	3.7	22126.5	3.6	22127.1	4.0
1.38 V	22127.3	4.1	22126.9	3.8	22126.9	3.8	22127.1	4.0
1.58 V	22127.8	4.4	22127.3	4.1	22127.0	3.9	22127.2	4.0
1.68 V	22127.8	4.4	22127.5	4.2	22127.1	4.0	22127.4	4.2
1.78 V	22127.9	4.5	22127.5	4.2	22127.1	4.0	22127.1	4.0
0.98 V	22127.3	4.1	22127.1	4.0	22126.8	3.8	22127.0	3.9
0.48 V	22126.7	3.7	22126.8	3.8	22126.3	3.4	22126.7	3.7
1.68 V	22127.7	4.4	22127.3	4.1	22127.1	4.0	22127.3	4.1

Table S7: Analysis of in situ Ir L_{III}-edge XANES data (for fitting procedure see method described above for reference materials). The missing point is from a scan for which evolution of oxygen bubbles prevented a usable spectrum being recorded.

Material→	$(Na_{0.33}Ce_{0.67})_2(Ir_{0.1}Ru_{0.9})_2O_7$		$(Na_{0.33}Ce_{0.67})_2(Ir_{0.5}Ru_{0.5})_2O_7$		$(Na_{0.33}Ce_{0.67})_2(Ir_{0.9}Ru_{0.1})_2O_7$		$(Na_{0.33}Ce_{0.67})_2Ir_2O_7$	
Applied potential *vs* RHE	Edge Position	Ir Oxidation State	Edge Position	Ir Oxidation State	Edge Position	Ir Oxidation State	Edge Position	Ir Oxidation State
0.98 V	11220.62	4.00	11220.47	3.91	11220.60	3.99	11220.57	3.97
1.38 V	11220.61	3.99	11220.51	3.93	11220.76	4.08	11220.79	4.10
1.58 V	11220.68	4.04	11220.57	3.97	11220.81	4.11	11220.89	4.16
1.68 V	11220.78	4.10	11220.64	4.01	11220.84	4.13	11220.97	4.21
1.78 V	11220.76	4.08	11220.64	4.01	-	-	11221.05	4.26
0.98 V	11220.58	3.98	11220.51	3.93	11220.35	3.84	11220.57	3.97
0.48 V	11220.51	3.93	11220.36	3.84	11220.85	4.14	11220.14	3.71
1.68 V	11220.74	4.07	11220.54	3.95	11220.60	3.99	11221.01	4.24

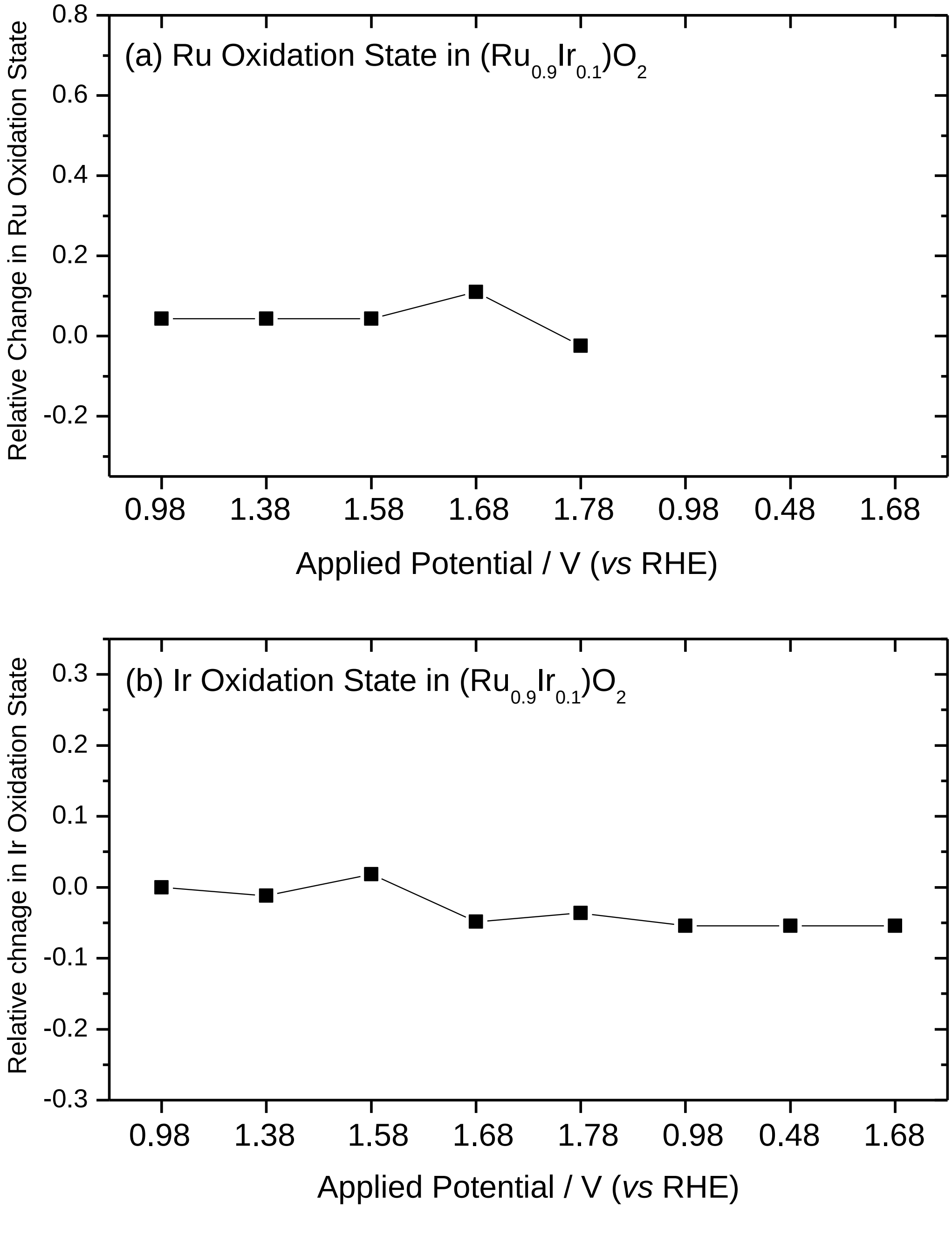

Figure S18: Results of* in situ *XANES analysis of reference materials (a) Ru oxidation state in commercial ($Ru_{0.9}Ir_{0.1}$)O_2 (b) Ir oxidation state in commercial ($Ru_{0.9}Ir_{0.1}$)O_2 Note in (a) the cycle was not completed due to the low shifts in oxidation state measured. The y-axes are plotted using the same scales as Figure 5.
